# Two cases of clear cell ovarian cancer in young patients

**Published:** 2016

**Authors:** Mohammad Ranaee, Shahla Yazdani, Seyyed Reza Modarres, Mahdieh Rajabi-Moghaddam

**Affiliations:** 1 Department of Pathology of Babol University of Medical Sciences, Babol, Iran.; 2 Department of Obstetrics and Gynecology Babol University of Medical Sciences, Babol, Iran.; 3 Department of Surgery Babol University of Medical Sciences, Babol, Iran.

**Keywords:** Ovarian neoplasms, Clear cell carcinoma, Young adult

## Abstract

**Background:**

Ovarian cancer is the most common cause of cancer death worldwide. Incidence of ovarian cancer is more common in postmenopausal women. Premenopausal onset is rare and the present study described two cases of ovarian clear cell tumors in young women.

**Case Presentation:**

The patients presented with pelvic mass which was confirmed by sonography and laparotomy and final diagnosis was made according to histologic examination. Both patients showed a solid mass with cystic components in adnexal areas and explorative laparotomy demonstrated extension of tumors to abdomen in both patients. The level of CA 125 increased in both patients. For both tumors, immunohistochemical stainings were positive for CK7 and CD15, but CK20 was negative.

**Conclusion:**

Although ovarian clear cell tumor is usually diagnosed in postmenopausal women but its diagnosis should be suspected in young women with pelvic mass.

Ovarian cancer is one of the most common gynecologic malignancies in different countries (1). It is the fifth most common cause of cancer death in women worldwide (1). A five-year survival of ovarian cancer patients is estimated to be 61%, in Iran (2). Incidence of ovarian cancer is more in postmenopausal women, with only 10% to 15% discovered in premenopausal patients (3). The lowest median age was seen in germ cell tumors (4) and the highest was observed in clear cell tumor. In Iranian population, the median age for diagnosis of ovarian cancer is between 30-59 years. Clear cell carcinoma of the ovary, especially in young patients, is a rare disorder. In Iran, the median age of ovarian clear cell carcinoma is 57 years old. We discuss two cases of ovarian clear cell carcinoma occurring in young patients.

## Case presentation

**Case 1.** A 29-year old, nulligravid woman presented with abdominal pain accompanied by dysuria and weight loss. She had prior history of pyelonephritis and suspected hydatidiform cyst. There were no other systemic symptoms. Her past family history was insignificant. The physical examination revealed a palpable mass in left lower abdomen with minimal abdominal distention. The CA125 level was 430.6 ng /mL. The CA19-9 level was 254.5 ng /mL, but AFP level was 1.16 ng /ml. A transvaginal ultrasound (TVS) was performed which demonstrated a heterogeneous solid cystic lesion, attached to left ovary, measuring 82×76 mm. 

**Figure 1:**
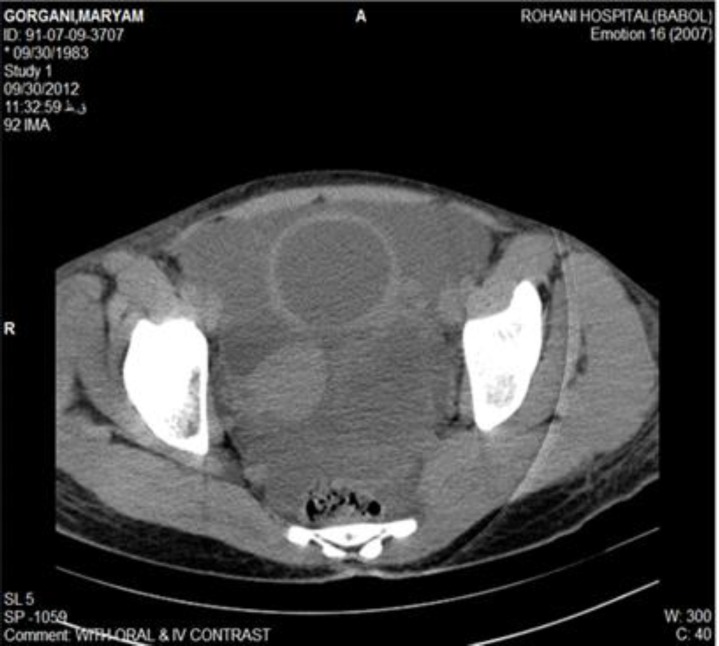
Abdominal CT-scan (with oral and intra-venous contrast

A preoperative CT showed a 90×80 mm, irregular, left adnexal solid-cystic mass and ascites accompanied by right pleural effusion and multiple cystic lesions in right hepatic lobe (figure 1). 

An exploratory laparotomy was performed. Intraoperatively, brown colored peritoneal fluid was seen. Left adnexal mass with solid and cystic components was present, filled with clear yellowish fluid and measured 100*100 mm. This lesion was attached to the bowel, gall-bladder and liver. 

Small nodules were seen over the right ovarian serosal surface. The uterine surface showed multiple serosal nodules. Multiple cystic lesions were seen over the right hepatic lobe surface. Total abdominal hysterectomy with bilateral salpingo-oopherectomy, omentectomy and appendectomy was performed. The partial hepatic resection was also done. The specimen was sent for histopathological examination. 

**Figure 2:**
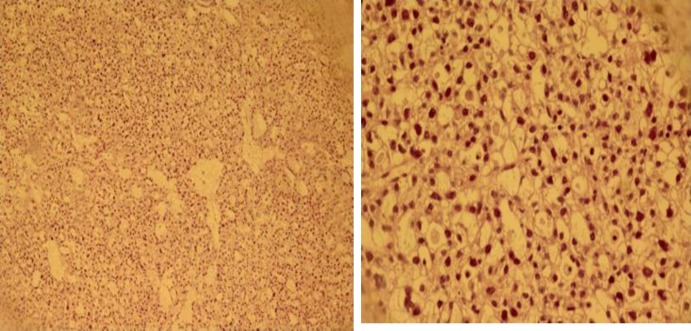
a: Nested pattern of clear cell tumor (100X), b: neoplastic cells with pleomorphic nuclei and clear cytoplasm (400X

Histopathology revealed ovarian tissue partially replaced by a neoplasic lesion composed of ovoid and polygonal pale eosinophilic to clear cells with distinct border and pleomorphic nuclei in glandular and micropapillary growth pattern. Multiple irregular follicles with cystic changes in some of them and some hobnail cells were also seen. The omentum and hepatic tissue were involved by tumor. Peritoneal fluid cytology revealed malignant cells (figure 2). 

**Figure 3:**
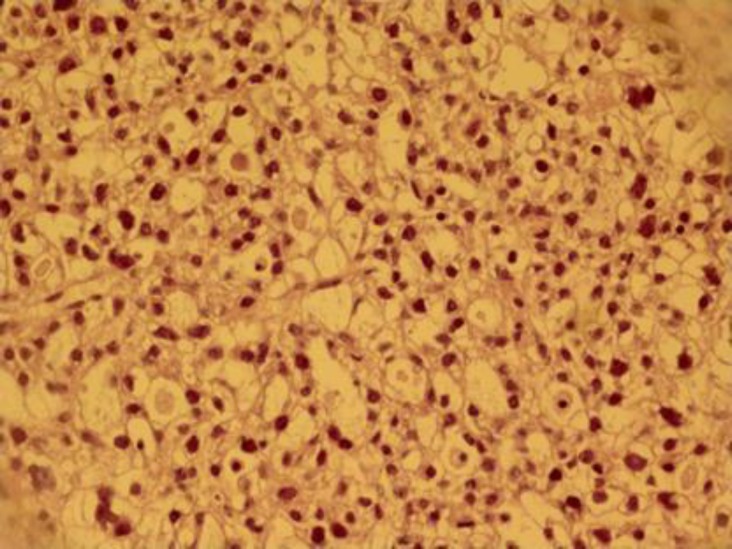
CD15 immunohistochemical staining

Immunohistochemical analysis of tumor was positive for CK7 and CD15, but CK20 was negative (figure 3). According to these findings, we made a diagnosis of ovarian clear cell carcinoma. Based on the TNM staging system for ovarian tumors, the patient was classified as stage IV. Postoperatively, oncologist recommended six cycles of chemotherapy with taxone and platinum with a 21-day interval, but the patient denied the treatment and unfortunately expired after a few months. 

**Case 2: **A 29-year old virgin woman presented only with increased abdominal circumference. Her past medical history showed muscularis dystrophia which started 10 years ago. There were no other systemic symptoms. The physical examination revealed a firm well- defined mass in left lower abdomen. The CA125 level was 586 ng /ml and AFP level was 22 ng /ml. A trans- abdominal ultrasound (TAS) was performed which demonstrated a well- defined cystic mass in pelvis, probably from the ovary, measuring 170×96 mm. A preoperative CT showed a 165×94 mm, cystic mass with solid component and mural nodules in pelvis that extended to abdomen. Ascites were also reported.

A unilateral salpingo-oophorectomy with partial omentectomy and appendectomy was performed. Left adnexal cystic mass with solid components was present, filled with brown fluid and measured 100×100 mm. The specimen was sent for histopathological examination. 

**Figure 4:**
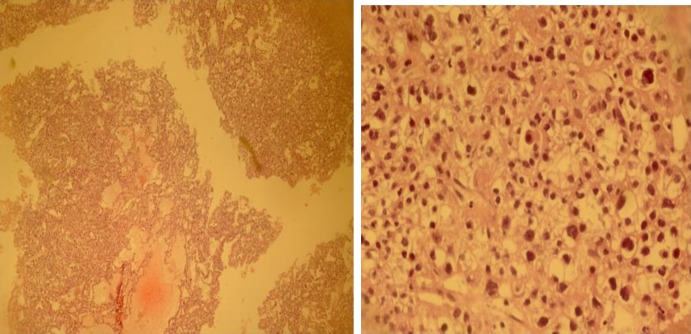
a: clear cell tumor (100X) b: tumoral cells with hobnail configuration (400X

Histopathology revealed ovarian tissue with a neoplasic lesion composed of nests of predominantly solid tumoral cells having abounded clear cytoplasm, distinct polygonal cellular border, enlarged vesicular moderate pleomorphic nuclei with mild mitotic figures and prominent nucleoli. In some foci, tubule- cystic structures, lined by tumoral cells with hobnail configuration, accompanied by short papillae and hyalinized cores is also seen (figure 4). Fallopian tube, omentum and appendix were free of tumor. Immunohistochemical analysis of tumor was focally strongly positive for CK7 and pan cytokeratin. 

According to histopathologic feature, CD15, CD30, EMA and CD117 were performed. CD15 and EMA were strongly positive, but other markers were negative. Based on these findings, we made a diagnosis of ovarian clear cell carcinoma. In reference to the FIGO grading system for ovarian tumors, the patient was classified as grade II (5). Total hysterectomy and right salpingo-oophorectomy was recommended, but patient denied this decision. 

## Discussion

In this case, we were involved with ovarian clear cell carcinomas in young women. Ovarian clear cell carcinoma has a distinct histopathologic feature, characterized by glycogen containing clear cells and “hobnail” cells.

Takano et al. (6), in a retrospective multicentre experience, evaluated the clinical characteristics and prognostic factors in patients with clear cell carcinoma (CCC) of the ovary. In their study, the mean age was 52.4 years. Five-year progression-free survival and overall survival was 84 and 88% stage I, 57 and 70% stage II, 25 and 33% in stage III and 0 and 0% in stage IV, respectively. In our cases, the age of patients was 29 years that is much lower than the mean age of patients in the abovementioned study. Also, our patients died after a few months of diagnosis that is in accordance to poor prognosis of this cancer.

In the study of Behbakht et al. (7) on the clinical characteristics of clear cell carcinoma of the ovary, the median age was 55 years (range 31–80 years). The age of our patients was even lower than the age of patients in that study. In their investigation, tumors were 60% stage I, 11% stage II, 20% stage III, and 9% stage IV. Our patients were in stage IV. Behbakht et al. stated that clear cell tumors of ovary frequently present at early stages; in contrast to this statement, our patients were end-stages.

The ovarian cancer cases consist of over 4% of all neoplasms in women (8). The incidence of ovarian cancer is quite variable worldwide. In developed countries, ovarian cancer is a common neoplasm (8). Ovarian cancer is the 8^th^ most common neoplasm in Iran (2). 

Ovarian cancer is a disease of postmenopausal women. The median age of the ovarian cancer is 49 years in Iran with the lowest and highest median age has been observed in germ cell tumors (4) and clear cell cancers, respectively (2). The median age of clear cell carcinoma is 57 years old in Iran. The incidence of clear cell carcinoma in female younger than 30 years of age is rare (2). 

In the recent decades, different independent factors have affected the incidence of ovarian cancer in Iran. Decreasing parity, increased use of oral contraceptive and change of diet, in favor of western diet have different impact (2). Additionally, sonography is available and inexpensive, resulting in the early detection and removal of a probable cancerous ovarian mass (2). Serum CA 125 levels and sonography are two useful screening tools for preoperative diagnosis and are also used for patient's follow-up (9, 10). These cases markedly elevated CA 125 levels at the time of presentation.

Cytoreductive surgery with adjuvant platinum based chemotherapy is performed for treatment. This tumor has a poor prognosis with a low survival and high recurrence rate (11, 12). With Stage IV respect to extremely poor prognosis of patients with ovarian clear cell carcinoma at stage IV, clinical implication of these case reports is that early diagnosis of such tumor of ovary could help in better survival of patients. Because there are no specific symptoms or signs for such tumor, we should be careful of the occurrence of such tumors even in young females, although this tumor is extremely rare in young patients.

To conclude, ovarian clear cell tumor in female less than 30 years of age is extremely rare and is a remote diagnosis. Misdiagnosis of such lesions may totally affect the treatment plan of ovarian tumor. Therefore, careful clinical and pathological examination is prudent.

## Citation:

Ranaee M, Yazdani S, Modarres SR, Rajabi-Moghaddam M. Two case reports of clear cell ovarian cancer in young patients. Caspian J Intern Med 2016; 7(3): 228-231.
